# How Learning to Read Changes the Listening Brain

**DOI:** 10.3389/fpsyg.2021.726882

**Published:** 2021-12-20

**Authors:** Linda Romanovska, Milene Bonte

**Affiliations:** Department of Cognitive Neuroscience, Faculty of Psychology and Neuroscience, Maastricht University, Maastricht, Netherlands

**Keywords:** reading development, dyslexia, audio-visual plasticity, reading-induced plasticity, dorsal and ventral reading networks

## Abstract

Reading acquisition reorganizes existing brain networks for speech and visual processing to form novel audio-visual language representations. This requires substantial cortical plasticity that is reflected in changes in brain activation and functional as well as structural connectivity between brain areas. The extent to which a child’s brain can accommodate these changes may underlie the high variability in reading outcome in both typical and dyslexic readers. In this review, we focus on reading-induced functional changes of the dorsal speech network in particular and discuss how its reciprocal interactions with the ventral reading network contributes to reading outcome. We discuss how the dynamic and intertwined development of both reading networks may be best captured by approaching reading from a skill learning perspective, using audio-visual learning paradigms and longitudinal designs to follow neuro-behavioral changes while children’s reading skills unfold.

## Introduction

Despite standardized curricula and teaching programs at school, children reach very different levels of reading fluency. Proficiency in reading determines personal achievement not only during primary and secondary education but also societal attainment later in life (UNESCO, 2006; Hudson et al., 2009; Livingston et al., 2018; Kortteinen et al., 2020). This especially affects the 5–10% of children with developmental dyslexia who struggle to acquire fluent reading skills despite adequate intellectual abilities and schooling opportunities (Blomert, 2005; Shaywitz and Shaywitz, 2008). Here we focus on neuro-behavioral processes characterizing the acquisition of early reading skills, and their relevance to explaining individual differences in children’s reading fluency at the level of visual words and pseudowords.

The acquisition of reading requires years of practice and is accompanied by a gradual re-shaping of existing dorsal spoken language and ventral visual brain networks into an integrated audio-visual reading network. Thus, when a child learns to read, striking changes occur in higher-order visual regions of the (left) ventral occipito-temporal cortex (vOTC) which becomes increasingly responsive to and specialized in written text perception (Maurer et al., 2006; Brem et al., 2009; Ben-Shachar et al., 2011; Dehaene-Lambertz et al., 2018). Furthermore, while forming associations between text and speech sounds, auditory/speech sensitive regions in the posterior superior temporal cortex (pSTC) become linked to these higher-order visual regions and start responding to written text in addition to spoken language (van Atteveldt et al., 2004, 2009, 2010; Froyen et al., 2009; Brennan et al., 2013; McNorgan et al., 2013, 2014; Kronschnabel et al., 2014; Bonte et al., 2017; Caffarra et al., 2021). The emergence of these audio-visual response characteristics arguably reflects a form of neural plasticity that is central to reading acquisition, with reduced or less automatic text-induced audio-visual linking in dyslexic readers and illiterates (Blomert, 2011; Dehaene et al., 2015). Individual differences in reading skills along a continuum from poor (dyslexic) to excellent readers, may thus scale with the capacity of the brain regions involved in auditory and visual perception to accommodate reading-induced changes. This may hold across largely different writing systems, with cultural variability mainly affecting the representational level at which written to spoken language associations are formed (Perfetti, 2003; Rueckl et al., 2015; Feng et al., 2020). Here we argue that understanding why some children thrive while others keep on struggling to read requires approaching reading-induced neuro-behavioral changes from a dynamic skill learning perspective, employing auditory and/or visual learning paradigms and multi-level longitudinal studies.

## Developmental Dyslexia

Developmental dyslexia provides a good model for investigating the role of the dorsal and ventral brain networks in reading development as most dyslexic readers show difficulties in handling the sound structure of spoken language (Snowling, 1980, 2013; Shaywitz et al., 1998; Goswami, 2003; Lyon et al., 2003) and in forming associations between (clusters of) letters and speech sounds (Blomert and Willems, 2010; Blomert, 2011; Kronschnabel et al., 2014). It remains debated whether the convergence of written to spoken language representations is a universal signature of proficient reading (Blomert, 2011; Rueckl et al., 2015), with a possible language-specific grain size of convergence (Ziegler and Goswami, 2005), or alternatively, is most relevant for explaining individual differences and dyslexia in orthographies with fairly regular letter-speech sound mappings, such as Dutch, German, or Hungarian, and less for languages with irregular mappings, such as English (Nash et al., 2016; Clayton and Hulme, 2017).

A major challenge in understanding dyslexia lies in its highly heterogeneous behavioral manifestation. Suggested causes include – but are not limited to – deficits in letter-speech sound integration (Snowling, 1980; Blomert, 2011), poorly specified and/or less categorical speech representations (Snowling, 1998; Serniclaes et al., 2004), impaired access to speech representations (Ramus and Szenkovits, 2008), impaired temporal sampling of speech (Goswami, 2011), inadequate implicit auditory regularity detection (Ahissar, 2007), impaired processing of brief sounds (Tallal and Piercy, 1973), visual dysfunctions (Bosse et al., 2007), or more general deficits in magnocellular functions (Livingstone et al., 1991), automation processes (Nicolson and Fawcett, 1999), or attentional mechanisms (Bosse et al., 2007; Shaywitz and Shaywitz, 2008; Vidyasagar and Pammer, 2010; Lobier et al., 2012). So far these different possibilities have been mostly studied in isolation and typically using cross-sectional experimental designs that may not have the sensitivity to reveal the underlying multifaceted and individually variable developmental dynamics. It is therefore promising that an increasing number of labs and research consortia have started longitudinal neuroimaging studies following children during different stages of reading development (van der Leij et al., 2013; Lyytinen et al., 2015; Wang et al., 2017, 2020; Dehaene-Lambertz et al., 2018; Vanderauwera et al., 2018; Chyl et al., 2019; Moulton et al., 2019; van de Walle de Ghelcke et al., 2020; Zuk et al., 2020, see Chyl et al., 2021 for a recent review of longitudinal neuroimaging studies on reading development and dyslexia). These studies are crucial to understanding how individual differences in reading trajectories and outcome can be positioned within the interactive development of the brain’s spoken and written language networks (Pugh et al., 2001; Sandak et al., 2004). Recent work has further highlighted that individual differences in reading outcomes are likely rooted in multiple genetic and environmental factors that interactively influence structural and functional brain changes while children learn and develop (Raschle et al., 2011; Wang et al., 2017; Yu et al., 2018b, 2020; Zuk et al., 2020). For example, a child with a parent or sibling with dyslexia has about 40–50% chance of also developing dyslexia. Neuro-behavioral risk factors, such as phonological processing difficulties, associated with this familial risk (Snowling and Melby-Lervåg, 2016) may be moderated by protective factors such as strong verbal reasoning, vocabulary and attention skills, or a positive self-concept (Cavalli et al., 2016; Haft et al., 2017). As a result of these complex interactive developmental processes, reading variability is continuous in nature (Pennington, 2006; van Bergen et al., 2014; Peters and Ansari, 2019). At the same time, dyslexia is typically diagnosed based on a specific cut-off, most commonly scoring 1,5 standard deviations below the age-group average on a battery of reading and/or spelling tests (American Psychiatric Association, DSM-5 Task Force, 2013). In clinical practice, such an arbitrary cut-off criterion is currently unavoidable, but at a scientific level, the variability and continuity in reading skills requires a shift from a dichotomous classification of reading as poor versus fluent toward a multi-deficit spectral view of reading (Pennington, 2006; van Bergen et al., 2014; Protopapas and Parrila, 2018; Peters and Ansari, 2019). Here, reading fluency is represented on a spectrum ranging from poor to fluent, with dyslexia lying on one end of the spectrum rather than being defined as a qualitatively discontinuous condition. This approach takes into account individual differences in reading proficiency observed across both poor and fluent readers (Aravena et al., 2013; Žarić et al., 2014; Fraga-González et al., 2015; Romanovska et al., 2021) and explains how the frequently reported comorbidity between developmental disorders, such as between dyslexia, dyscalculia, and attention deficit hyperactivity disorder (ADHD), may result from shared neurobiological and/or environmental risk factors (Pennington, 2006; Landerl and Moll, 2010; van Bergen et al., 2014; Peters and Ansari, 2019).

## Speech Perception in the Dorsal Language Network

**Figure 1:**
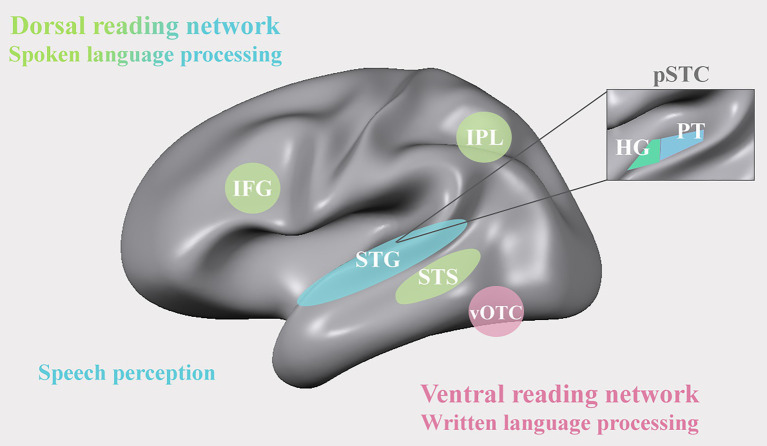
A representation of the dorsal (green) and ventral (pink) reading networks of the brain’s audio-visual reading network. IFG: inferior frontal gyrus; IPL: inferior parietal lobe; STG: superior temporal gyrus; STS: superior temporal sulcus; vOTC: ventral occipito-temporal cortex; pSTC: posterior superior temporal cortex; HG: Heschl’s gyrus; PT: planum temporale.

By the time children start to learn to read, neural functions for speech perception and production have already gone through several years of functional refinement. Spoken language functions thus form a linguistic basis for reading acquisition both from a phylogenetic and an ontogenetic perspective (Dehaene et al., 2015). Since the first neuroimaging findings in the 1990s, numerous studies have been designed with the aim to delineate the brain’s spoken language system. One of the pioneering studies in this domain showed that listening to speech elicits extensive and bilateral activation in the superior temporal cortex (STC), including primary areas on Heschl’s gyrus (HG), the planum temporale (PT), and along the superior temporal gyrus (STG) and superior temporal sulcus (STS; see Figure 1; Binder et al., 1994). Building on this work, advances in, among others, functional magnetic resonance imaging (fMRI) and Electrocorticography (EGoG) methodology have enabled delineating a more fine-grained functional architecture of speech sound representations in the superior temporal cortex (Chang et al., 2010; Mesgarani et al., 2014; Leonard et al., 2016). One relevant new insight emerging from this work is the finding that the auditory representations of speech along the posterior and lateral STG are not restricted to low-level acoustic-phonetic speech features (Jäncke et al., 2002; Obleser and Eisner, 2009), but include higher-order perceptual levels of representation that are strongly modulated by a listener’s behavioral goals, learning and contextual information (Formisano et al., 2008; Bonte et al., 2009, 2014; Mesgarani and Chang, 2012; Rutten et al., 2019; Yi et al., 2019; Levy and Wilson, 2020). Most interesting with respect to reading development is the observation of perception-related shifts in the auditory cortical representation of speech resulting from visual presentation of text (Bonte et al., 2017) and other types of multisensory context information, including videos of a speaker articulating words or pseudowords (Kilian-Hütten et al., 2011; Ozker et al., 2017, 2018). While it remains debated whether and how learning to read changes the representation of speech (Dehaene et al., 2015; Mitterer and Reinisch, 2015), the modulatory effect of audio-visual mappings between text and spoken language does suggest reading-induced plasticity at the level of the auditory cortex (Bonte et al., 2017; see also Karipidis et al., 2017, 2018; Joo et al., 2021).

Further support for a key role of the superior temporal cortex in learning to read comes from developmental neuroimaging studies showing that the strength and/or extent of speech evoked responses in this region is associated with children’s reading level (Parviainen et al., 2011; Brennan et al., 2013; Conant et al., 2014; Lohvansuu et al., 2018), and phonological skills (Turkeltaub et al., 2003; Conant et al., 2014; Bonte et al., 2016; Randazzo et al., 2019). Moreover, children with dyslexia (Schulte-Körne et al., 1998; Bonte and Blomert, 2004; Frey et al., 2019; Schaadt and Männel, 2019; Gu and Bi, 2020; Virtala et al., 2020), or at familial risk for developing dyslexia (Vandermosten et al., 2020) may show reduced or less discriminable auditory cortical responses to speech. Such functional changes may be the result of less efficient speech sound learning during early development. Indeed, a reduced, or a slower build-up of, sensitivity to statistical regularities in speech sound structures has been observed in adults (Noordenbos et al., 2013; Schmalz et al., 2017; Zhang et al., 2021) and children (Bonte et al., 2007; Gabay et al., 2015) with dyslexia. Evidence further suggests that atypicalities in brain responses to speech (Vandermosten et al., 2020), and to basic sound features (Hakvoort et al., 2014) may be associated with a familial risk for dyslexia without being predictive of children’s later reading outcomes. Thus, atypicalities in auditory cortical responses to speech, together with commonly observed phonological processing difficulties in children at familial risk for dyslexia (Snowling and Melby-Lervåg, 2016), likely present a vulnerability or risk factor for reading problems that will lead to dyslexia if not mitigated by protective factors. This phonological risk factor may also show in anatomical characteristics of speech sensitive superior temporal cortex (STC). In fact, there is a long tradition of relating morphological variability of the PT to language dysfunctions in dyslexia (Geschwind and Levitsky, 1968; Galaburda et al., 1985; Galaburda, 1989; Leonard et al., 2006) as well as to inter-individual variability in auditory and language skills (Golestani et al., 2011).

A relation between reading development and morphological characteristics of the superior temporal cortex can be located within a more general pattern of protracted, experience-related changes in pSTC morphology, which have been observed to continue well into the third decade of life, particularly in the left hemisphere (Giedd et al., 1999; Sowell et al., 2003; Gogtay et al., 2004). Similarly, while the global signature of speech evoked STC responses is in place in infancy (Dehaene-Lambertz and Pena, 2001), its functional characteristics continue to change well beyond primary school years (Sharma et al., 1997; Pang and Taylor, 2000; Bonte and Blomert, 2004; Brauer et al., 2008; Bonte et al., 2013; Chyl et al., 2017). Such an extended developmental time course may allow a prolonged process of functional specialization during which auditory and visual language input contributes to the shaping and fine-tuning of pSTC brain circuitry (Johnson, 2001, 2011; Werker and Hensch, 2015). Indeed, a recent study exploring grey matter volume in 8-year-old children of varying reading fluency found that right STG grey matter volume differentiates fluent from dysfluent readers, with the former group showing higher grey matter volume in this region compared to the latter group (Martins et al., 2021). So far, however, there is no unequivocal evidence linking morphological pSTC features to its functional characteristics or to individual children’s language skills. Early research specifically reported a hemispheric asymmetry of the PT region, with the left PT covering a larger surface area compared to the right PT in 65% of a sample of 100 brains (Geschwind and Levitsky, 1968). Soon after, studies emerged suggesting that the same asymmetry is not present in readers with dyslexia who instead were found to have more symmetrical PT areas in both hemispheres or to show the opposite, right-ward, asymmetry (Galaburda et al., 1985; Galaburda, 1989). It was therefore argued that reading difficulties in dyslexia may be associated with morphological differences of the PT. However, the finding of a different PT asymmetry in readers with dyslexia has not been systematically replicated (Leonard et al., 1993, 2006; Schultz et al., 1994; Beaton, 1997; Carrion-Castillo et al., 2020) and may instead depend on more general factors, such as gender (Altarelli et al., 2014), family history of dyslexia (Vanderauwera et al., 2018), handedness (Beaton, 1997), or methodological discrepancies in the anatomical criteria used to delineate the PT (Ramus et al., 2018). Thus, similar to behavioral and functional STC signatures of phonological processing difficulties, differences in PT morphology may reflect a neurobiological risk factor for later reading problems rather than characterizing all individuals with developmental dyslexia.

## Audio-Visual Processing in the Dorsal and Ventral Reading Networks

The protracted functional and morphological development of the pSTC in the posterior dorsal network may be key to the gradual build-up of neural associations between visual symbols and corresponding spoken language representations and, on a more general level, the strong bi-directional influences between reading and spoken language development (Morais et al., 1979; Perfetti et al., 1987; Sandak et al., 2004; Blomert, 2011; Rueckl et al., 2015). Evidence of a gradual strengthening of audio-visual associations throughout the first years of reading development – extending well beyond the initial phase of learning to map letter(s) to their corresponding speech sound(s) – comes from EEG studies employing audio-visual oddball paradigms. These studies have found that the neural time-window of audio-visual integration changes from later to earlier (Froyen et al., 2009; Žarić et al., 2014) and becomes narrower/more time-sensitive (Žarić et al., 2014) over the course of (reading) development. Moreover, the latency of integrative letter-speech sound responses has been found to deviate in dyslexic compared to typical readers (Froyen et al., 2011; Žarić et al., 2014; Jones et al., 2016; Moll et al., 2016) with a speeding up of these responses after 6 months of intensive letter-speech sound training in dyslexic children (Žarić et al., 2015). Suggestive evidence for a direct influence of visual text on pSTC responses to speech comes from the observation that pSTC activation increases in response to matching (congruent) compared to non-matching (incongruent) letter-speech sound pairs and speech alone (Raij et al., 2000; van Atteveldt et al., 2004; Blau et al., 2010; van Atteveldt and Ansari, 2014; Karipidis et al., 2017). Furthermore, these cross-modal modulations in the STC were found to scale with phonological skills (McNorgan et al., 2013) and reading experience (McNorgan et al., 2014) in typical readers, and to be reduced in dyslexic readers (Blau et al., 2009, 2010; Kronschnabel et al., 2014; Ye et al., 2017). Similarly, we recently observed that cortical activation in bilateral STG in response to paired text and ambiguous speech sound stimuli correlates with children’s letter-speech sound mapping fluency (Romanovska et al., 2021).

While the pSTC seems to be especially relevant for the processing of already learnt letter-speech sound associations (van Atteveldt et al., 2004; Blau et al., 2010), the inferior parietal lobe (IPL) may mediate the initial establishment of these associations (Hashimoto and Sakai, 2004; Booth et al., 2007; Bonte et al., 2017; Wise Younger et al., 2018). In typically reading adults, text-induced shifts in superior temporal cortical responses to ambiguous speech sounds seem to be “installed” via functionally correlated activity in the IPL (Bonte et al., 2017), and learning of novel symbol-sound mappings is modulated by parietotemporal brain stimulation (Wise Younger et al., 2018). Furthermore, continued reading development in children is associated with a reduction in IPL activation in response to text and audio-visual phonological processing, as well as with a reduction in reading-related IPL to vOTC connectivity (Wise Younger et al., 2017; Dehaene-Lambertz et al., 2018; Yu et al., 2018a; Moulton et al., 2019). Studies comparing brain activation within this region between readers with and without dyslexia have found reduced IPL activation in both, adults and children with dyslexia (Hoeft et al., 2007; Richlan et al., 2009; Paz-Alonso et al., 2018), with a possibly more pronounced group difference in children (Richlan et al., 2011). Next to a specific contribution to (the learning of) letter-speech sound mapping, the IPL has been associated with other linguistic functions including semantic processing (Shaywitz and Shaywitz, 2008; Paz-Alonso et al., 2018) as well as with more general cognitive functions including visual attention (Vidyasagar, 1999; Saalmann et al., 2007). The involvement of the IPL in both letter-speech sound mapping and visual attention is interesting also with respect to the frequent co-occurrence of dyslexia and, especially the inattentive subtype of, ADHD (Greven et al., 2011; Hendren et al., 2018; Plourde et al., 2018).

**Figure 2:**
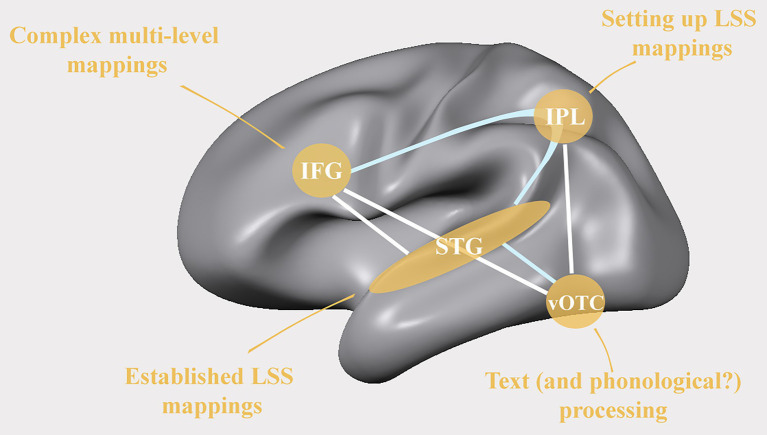
Areas where reduced cortical activation has been reported in readers with dyslexia alongside frequently observed differences in functional (white lines) and structural (light blue lines) connectivity between readers with and without dyslexia. IFG: inferior frontal gyrus; IPL: inferior parietal lobe; STG: superior temporal gyrus; vOTC: ventral occipito-temporal cortex; LSS: letter-speech sound.

The anterior part of the dorsal network houses the inferior frontal gyrus (IFG) which is increasingly recruited while beginning readers improve their reading ability and phonological skills (Turkeltaub et al., 2003). Functional connectivity between IFG and IPL has been associated with phonological processing skills during early reading development (Yu et al., 2018a), while functional connectivity between IFG and STG has been found to correlate with reading fluency measures and to be reduced in dyslexic readers (Figure 2; Schurz et al., 2015). Furthermore, studies have shown reduced activation in the left IFG (Cao et al., 2006; Richlan et al., 2009; Richlan, 2012, 2019) but increased activation in left pre-motor regions in dyslexic compared to typical readers (Richlan et al., 2010; Wimmer et al., 2010). The increased left-premotor activation is interesting as, next to the suggested compensatory function of the right IFG in dyslexic readers (Hoeft et al., 2011), it may provide a window on possible alternative reading strategies or paths to improving learning outcomes via sensorimotor training, including, for example, the active pronunciation, or writing of letters (Torres et al., 2021). Within the developing reading network, the IFG may be involved in the learning of novel audio-visual associations (Hein et al., 2007), phonologic-orthographic regularity of words (Pugh et al., 1997), and semantic and phonological processing of written and spoken words (Fiez, 1997; Poldrack et al., 1999; Booth et al., 2001; Burton, 2001; Turkeltaub et al., 2003; Sandak et al., 2004). These different levels of analysis are compatible with a more general role of the left IFG in unifying different types of linguistic and non-linguistic information into multi-level integrated language representations (Hagoort, 2005).

A core area for developing fluent reading in the ventral reading network is the putative visual word form area (VWFA) within the left vOTC. This area has been shown to become increasingly specialized for text over the course of reading development (Maurer et al., 2006; Brem et al., 2009; Ben-Shachar et al., 2011; Dehaene-Lambertz et al., 2018) and to be less active in dyslexic readers (Figure 2; Paulesu, 2001; Hoeft et al., 2007; Richlan et al., 2009; Wimmer et al., 2010; Dehaene and Cohen, 2011). The central function of this specific occipito-temporal region in written text processing most likely relates to its close functional interaction with regions in the dorsal language network, including the STS, pSTG, IPL, and IFG (Richlan, 2012; Monzalvo and Dehaene-Lambertz, 2013; Schurz et al., 2015; Yu et al., 2018a). Accordingly, the functional specialization of the left vOTC is thought to be shaped by communication via direct white matter connections to these key speech processing areas (Hannagan et al., 2015; Saygin et al., 2016; Moulton et al., 2019). Indeed, in literate participants in alphabetic languages, activation in this region has been linked to categorical perception of phonemes (Conant et al., 2014), phonological processing (Romanovska et al., 2021), and to be modulated by audio-visual speech-text stimuli (McNorgan and Booth, 2015). Moreover, developmental studies report more overlap in activation in response to both, auditory and visual word stimuli in the vOTC and STG in children compared to adults (Booth et al., 2001), with a gradual transition from multi-modal to primarily unimodal processing with continued (reading) development (Church et al., 2008). Its lasting functioning as a multi-modal language area is also indicated by the involvement of the left vOTC during braille reading or reading via soundscapes in the congenitally blind (Büchel et al., 1998; Burton et al., 2002; Reich et al., 2011; Striem-Amit et al., 2012) and its responsiveness to both (braille) reading and grammatical processing of spoken sentences in congenitally blind braille readers but not in sighted readers (Kim et al., 2017).

One important open question is the extent to which the commonly observed reduced recruitment of regions within the dorsal and ventral reading networks in dyslexic readers constitute risk and/or protective factors in the etiology of dyslexia, or alternatively reflect consequences of a history of reading problems (see e.g., Huettig et al., 2018). Longitudinal studies following children with/without family risk of dyslexia over the course of reading development will be important to disentangle the contribution of each of these factors to explaining individual differences. Available evidence suggests that pre-readers categorized at high versus low family risk for developing dyslexia, show activation differences in similar brain regions as dyslexic versus typical readers (Figure 2). These include reduced activity in key spoken language and reading networks (Debska et al., 2016), and more specifically in the left vOTC (Plewko et al., 2018), and (letter and) speech sensitive left STC (Maurer et al., 2003; Raschle et al., 2012; Plewko et al., 2018) alongside reading outcome related group differences in white matter diffusivity between left ventral visual and frontal regions (Vandermosten et al., 2015; Vanderauwera et al., 2017). Specifically, family risk was found to be associated with a reduced distinctiveness of STC speech representations in 7 to 8-year-old children (Vandermosten et al., 2020), and reduced left temporo-parietal cortical activity during phonological processing in pre-readers (Yu et al., 2018a). Neither of these diminished speech/phonology evoked functional responses was found to be predictive of children’s later reading problems. On the other hand, brain activation of the right IFG during phonological processing and vowel perception tasks (Leppänen et al., 2011; Yu et al., 2020) as well as changes in white matter diffusivity in tracts connecting the dorsal and ventral reading systems (Wang et al., 2017) in at-risk children who do versus do not develop reading difficulties have been reported to differ from children without familial risk. The nature of developmental changes in network dynamics and compensatory mechanisms children develop to aid reading, will likely differ depending on family risk of dyslexia, as well as additional social and environmental risk and protective factors.

## Developmental Dynamics of the Dorsal and Ventral Reading Networks

**Figure 3:**
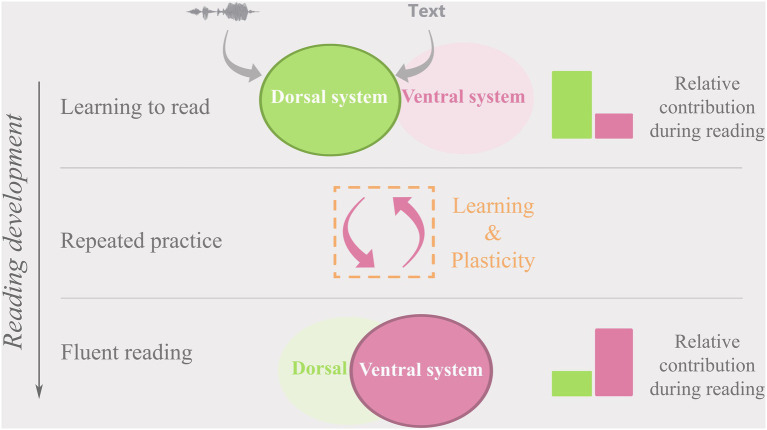
A representation of the relative dorsal (green) and ventral (pink) cortical reading network contribution over the course of reading development.

When learning to read, children initially rely mostly on the dorsal network (Figure 3, top row; Booth et al., 2001; Pugh et al., 2001; Turkeltaub et al., 2003; Sandak et al., 2004), presumably employing the IPL in particular to map letters and eventually letter strings onto corresponding speech sounds (Shaywitz and Shaywitz, 2008; Grainger and Ziegler, 2011; Moulton et al., 2019). With continued practice, both ventral and dorsal networks are shaped by the incremental development of phonological awareness and integrated representations of orthographic, phonological, and semantic features of words (Pugh et al., 2013). Effortful letter-for-letter reading and emerging phonological and orthographic knowledge create and strengthen functional (Schlaggar and McCandliss, 2007; Price and Devlin, 2011; Dehaene et al., 2015; Yu et al., 2018a; Moulton et al., 2019) and structural (Yeatman et al., 2012; Vandermosten et al., 2012b; Gullick and Booth, 2014; Myers et al., 2014; Moulton et al., 2019) links between the dorsal and ventral systems. Ample evidence suggests that this leads to a gradual re-shaping of dedicated areas for visual text processing in the left vOTC (Pugh et al., 2001; Maurer et al., 2006, 2008; Brem et al., 2009, 2010; Price and Devlin, 2011; Fraga González et al., 2014, 2016, 2017; Lochy et al., 2016; Karipidis et al., 2017, 2018; Pleisch et al., 2019).

While there is discussion about the extent to which reading acquisition also changes auditory speech representations (Dehaene et al., 2015; Mitterer and Reinisch, 2015; Bonte et al., 2017), recent neuroimaging findings in children indeed suggest these types of changes in the STG (Karipidis et al., 2017, 2018; Joo et al., 2021). For example, 7–12-year-old typical readers have been found to automatically engage the left STG while processing visually presented words, with stronger left STG responses in better readers (Joo et al., 2021). Research employing artificial script learning paradigms in pre-readers has shown higher right STG activation in response to trained versus untrained letter-symbol pairs in children who were faster in learning these associations (Karipidis et al., 2017). Moreover, left PT activation has been found to differentiate future fluent and poor readers, with an increased PT activation in response to congruent compared to incongruent letter-symbol pairs in future fluent readers and a trend toward the opposite response pattern in future poor readers (Karipidis et al., 2018).

Based on longitudinal evidence, the strength of functional connectivity between the IPL and vOTC is suggested to be key to successful audio-visual integration of letters and speech sounds during initial reading development and to shaping the left vOTC for automatized, fluent reading (Wise Younger et al., 2017; Yu et al., 2018a). Once fluent reading is achieved, the ventral network ensures rapid and automatized recognition and processing of text and becomes the dominant system used for reading (Figure 3, bottom row; Shaywitz et al., 2002; McCandliss et al., 2003; Sandak et al., 2004; Cohen and Dehaene, 2009). While the contribution of the dorsal network to fluent reading at this stage may be reduced, areas within the network continue to be employed especially for reading difficult, irregular words, or novel pseudowords (Pugh et al., 2001; Simos, 2002). In line with the proposed developmental shift to predominant reliance on the ventral system in fluent readers, increased connectivity between the IPL and vOTC is linked to better reading fluency and phonological skills in 5 to 6-year-olds (Yu et al., 2018a; Moulton et al., 2019), while decreased connectivity between these areas has been associated with larger gains in reading fluency in 8 to 14-year-old children (Wise Younger et al., 2017). These findings suggest a dynamic relationship between the dorsal and ventral networks where the strength of their inter-connectedness follows a non-linear inverted-u-trajectory with reading development (see also skill learning perspective below). The developmental changes in functional connectivity are paralleled by changes in diffusivity in the white matter tracts connecting the key nodes of the dorsal and ventral reading networks (Yeatman et al., 2012; Myers et al., 2014; Vanderauwera et al., 2018) the developmental trajectories of which may be different for good versus poor readers (Yeatman et al., 2012).

## Skill Learning and Non-Linear Developmental Changes

When acquiring a new skill, children make use of both, active, explicit strategies as well as implicit, statistical/associative learning (Shrager and Siegler, 1998; Siegler, 2005; Siegler and Araya, 2005). During the initial stages of learning, the active strategies are relied upon the most, helping to establish rules necessary to acquire a skill (Crowley et al., 1997; Karni et al., 1998). Continued practice serves as input for the implicit learning mechanisms that are mainly used during a consolidation phase involving the mastering of a given skill (Crowley et al., 1997; Shrager and Siegler, 1998; Siegler and Araya, 2005). Models of skill learning further predict that, at the brain level, learning follows an initial phase of expansion (e.g., an increase in regional activity or cortical maps) with a subsequent renormalization (e.g., a reduction of regional activity or cortical maps; Wenger et al., 2017; Lövdén et al., 2020). While these models are mostly based on perceptual, motor skill learning (Karni et al., 1998; Wenger et al., 2017; Lövdén et al., 2020), and math learning (Shrager and Siegler, 1998; Siegler, 2005; Siegler and Araya, 2005), reading acquisition similarly involves an initial phase of explicit learning of letter-speech sound mappings followed by a slower consolidation phase involving the development of reading fluency with practice and experience. In fact, several neuroimaging studies have suggested that the acquisition of reading during primary school is accompanied by non-linear inverted-u-type changes in visual and auditory cortical responses to text and audio-visual letter-speech sound stimuli (Maurer et al., 2006, 2008, 2011; Price and Devlin, 2011; Fraga González et al., 2017; Dehaene-Lambertz et al., 2018; Fraga-Gonzalez et al., 2021). Similar non-linear changes have also been reported in connectivity between the reading networks. A longitudinal study investigating changes in structural connectivity between key areas of the ventral and dorsal reading networks in children aged 5 showed an increase in connectivity between the VWFA and left IPL during the first year of reading instruction, that correlated with reading ability (Moulton et al., 2019). Longitudinal studies of functional connectivity changes in 5-to-8-year-olds, have reported a developmental increase in connectivity strength between the left IPL and lateral posterior occipito-temporal cortex in children with above-average gains in phonological processing, with children who had below-average gains showing the opposite pattern (Yu et al., 2018a). By comparison, in older, 8-14-year-old, children a longitudinal decrease in functional connectivity between the IPL and fusiform gyrus was associated with reading gains. This reduction in connectivity was observed in children who showed the largest improvement in reading across sessions (Wise Younger et al., 2017). We therefore hypothesize that, next to regional brain changes, learning to read involves dynamic trajectories of functional connectivity, starting from less reliable dorsal-to-ventral connectivity at the onset of reading instruction (i.e., no robust associations between written and spoken language), to an increase (expansion) in connectivity strength with initial reading acquisition (e.g., Yu et al., 2018a; Moulton et al., 2019), followed by a decrease in (renormalization) connectivity with repeated practice (e.g., Wise Younger et al., 2017). Once reading has become fully automatized, fast and fluent reading is mainly taken over by the ventral system (Pugh et al., 2001; Sandak et al., 2004).

Changes in cortical activation in the regions of the ventral and dorsal reading networks as well as the connectivity patterns between these areas could reflect their gradual specialization for reading. Thus, after initially establishing broad and varied links between written and spoken language, similar to model of sensory and motor skill learning (Lövdén et al., 2020), only the most efficient links may be reinforced through repeated reading practice. This selection process may shape the specialization and consolidation of local representations of visual text in the vOTC (Maurer et al., 2006; Brem et al., 2009; Ben-Shachar et al., 2011; Dehaene-Lambertz et al., 2018) and of text-to-spoken language mappings in the pSTC (Froyen et al., 2009; Brennan et al., 2013; McNorgan et al., 2014; Caffarra et al., 2021). Local specialization, in turn, may be characterized by narrowing of response properties – that is, increased sensitivity to text compared to other visual stimuli (Dehaene-Lambertz et al., 2018) – and increased local processing speed (e.g., within the vOTC Johnson, 2001). These local and interregional developmental changes in the reading network may follow non-linear inverted-u-type trajectories (Froyen et al., 2009; Fraga González et al., 2017; Fraga-Gonzalez et al., 2021), but also other types of (non)linear trajectories (Bonte et al., 2016; Dehaene-Lambertz et al., 2018).

Individual differences in the time course of reading development will inevitably affect the timing and pattern of changes in the dorsal and ventral reading networks. The time it takes to become a fluent reader is influenced by (but not limited to) genetic (Hawke et al., 2006; Keenan et al., 2006; Friend et al., 2008) and socio-economic factors (Noble et al., 2006a,b; Aikens and Barbarin, 2008). Especially individuals with (familial risk of) dyslexia may require an extended period for speech structure and audio-visual learning (e.g., Karipidis et al., 2018; Zhang et al., 2021). A longitudinal investigation of children with and without dyslexia showed delayed development of functional connectivity between vOTC and IFG between ages 6 to 8 in dyslexic compared to age-matched typical readers, reaching the same level of connectivity by age 12 (Morken et al., 2017). Aberrant functional connectivity between vOTC and the dorsal network has also been reported in cross-sectional studies, with dyslexic children showing less robust connectivity between the left vOTC, IFG, and IPL (Figure 2; van der Mark et al., 2011; Finn et al., 2014; Schurz et al., 2015). The connectivity patterns in these studies reveal alternate functional connectivity between the vOTC and the dorsal system, as well as connectivity to right hemisphere areas, arguably as a result of differences in the developmental trajectories in poor and fluent readers. Developmental changes in functional connectivity between the IPL and left vOTC may be key for a successful switch to automatized, ventral processing of text in typical readers (Wise Younger et al., 2017). However, dyslexic readers and at-risk pre-readers may develop different functional and structural (Steinbrink et al., 2008; Rimrodt et al., 2010; Vandermosten et al., 2012a, 2015, 2017; Cui et al., 2016; Langer et al., 2017; Vanderauwera et al., 2017) connectivity patterns between the ventral and dorsal reading networks, potentially as a compensatory reaction to difficulties with quick, automatized text processing in the vOTC.

## Investigating Text-Speech Sound Learning Mechanisms

In line with the idea that the brain’s reading network is formed through the association of written to spoken language representations, our understanding of individual differences in reading development will benefit from a detailed understanding of learning processes underlying the formation of these associations. Studies investigating dyslexia intervention targeting letter-speech sound automatization, have shown improvements in reading outcome and in brain responses associated with letter-speech sound integration following intervention (Žarić et al., 2015; Fraga González et al., 2016, 2017). However, typically used outcome measures of letter-speech sound integration (e.g., (in)congruency effects of learnt associations) yield variable neuro-behavioral differences in group comparisons of typical readers compared to dyslexic readers at different ages, and pre-readers at high versus low familial risk (Blau et al., 2010; Richlan et al., 2011; Žarić et al., 2014; Karipidis et al., 2018; Plewko et al., 2018). It is difficult to assess children’s underlying letter-speech sound mappings in these paradigms in a way that is not biased by context variables, such as task strategies and (self-beliefs regarding) the ability to perform the experimental task. Interestingly, longitudinal behavioral evidence suggests that pre-literate children’s ability to learn letter-speech sound associations – rather than their current knowledge of these associations – permits predicting individual differences in early reading skills (Horbach et al., 2015, 2018).

A promising platform to investigate audio-visual learning mechanisms can be found in artificial symbol – (speech) sound training and phonetic recalibration paradigms. Artificial symbol – sound training involves mapping known speech sounds onto novel visual symbols, thus directly targeting reading-related learning skills. Despite the observed association between pre-literate children’s symbol-sound learning performance and their early reading skills (Horbach et al., 2015, 2018), no group differences in the overall ability to learn novel letter-speech sound pairs have been observed between 7- to 11-year-old children with dyslexia (Aravena et al., 2013; Law, 2018) and at-risk pre-readers (Karipidis et al., 2018) compared to their age-matched peers. Group differences have been found to emerge under time constraints (i.e., rapid naming of the letter-speech sound pairs; Aravena et al., 2013 but see Law, 2018) and when the newly learnt letter-speech sound mappings needed to be applied to another task (e.g., reading names of familiar objects using the artificial script; Aravena et al., 2013; Karipidis et al., 2018; Law, 2018). Also, the use of these mappings for decoding, including blending phonemes into syllables and word reading, has been found to predict children’s future reading problems (Gellert and Elbro, 2017). Together, these findings suggest that reading problems may especially occur if a child faces difficulties in consolidating or automatizing letter-speech sound mappings rather than in creating these mappings in the first place (Blomert and Willems, 2010; Blomert, 2011; Kronschnabel et al., 2014).

Another paradigm that enables examining perceptual mechanisms associated with short-term audio-visual learning is phonetic recalibration (also “perceptual learning,” Samuel and Kraljic, 2009; Vroomen and Baart., 2012). Recalibration refers to a shift in an individual’s perception of ambiguous speech induced by the presentation of disambiguating visual input, such as lip-read speech (Bertelson et al., 2003; Vroomen and Baart., 2012), spoken word context (Norris et al., 2003), overt speech articulation (Scott, 2016), or text (Bonte et al., 2017; Keetels et al., 2018; Romanovska et al., 2019). In the classical paradigm, an ambiguous speech sound, e.g., /a?a/ midway between /aba/ and /ada/ is combined with a disambiguating video of a speaker articulating “aba” or “ada.” The subsequent perception of the ambiguous speech sound in auditory-only trials is temporarily biased in the direction of the video – that is, it will be perceived as /aba/ following an “aba” video and as /ada/ following an “ada” video. This perceptual shift is accompanied by a measurable shift in fMRI activation patterns in early and higher-order auditory cortex (Kilian-Hütten et al., 2011). Namely, multi-voxel pattern analysis of left PT and HG activity, enabled to significantly distinguish whether, on a given trial, participants perceived the ambiguous /a?a/ sound as either /aba/ or /ada/. In other words, the same ambiguous /a?a/ sound was represented differently depending on the disambiguating video it had been coupled with. This shift indicates that the two modalities have been successfully combined and a new audio-visual association created.

An alternative to the classical recalibration paradigm – text-based recalibration – employs text as the disambiguating visual information, tapping into the mechanisms of reading-induced audio-visual plasticity. An fMRI study exploring text-based recalibration in typically reading adults found that text-induced perceptual shifts in the auditory cortical representations of ambiguous speech is mediated by the bilateral IPL (Bonte et al., 2017). Behavioral evidence has further suggested an absence of this text-induced perceptual shift in adult dyslexic readers (Keetels et al., 2018) while lip-read information was found to yield similar shifts in dyslexic and fluent readers (Baart et al., 2012; Keetels et al., 2018). Surprisingly, 8 year-old dyslexic children instead were found to show comparable text-based recalibration to their typically reading peers (Romanovska et al., 2019), emphasizing the importance of studying audio-visual learning processes across multiple age groups. At the same time, we found different cortical activation patterns accompanying these comparable behavioral text-based recalibration effects in children with and without dyslexia (Romanovska et al., 2021). Children with dyslexia showed less vOTC activation during audio-visual integration of letters and ambiguous speech compared to typically reading children. Moreover, cortical activation within this region was correlated with individual differences in reading fluency and phonological processing across groups. Additionally, across groups, higher bilateral STG activation was associated with less fluent letter-speech sound integration (Romanovska et al., 2021). These findings point to a relative difference in fluent versus less fluent reader’s reliance on brain areas in the ventral and dorsal reading networks. Because the interplay between both networks is still being refined during initial reading development, less fluent readers may engage the dorsal reading network to a higher extent to successfully map letters and speech sounds. Once a more fixed and mature connectivity pattern has been established, group differences may emerge as a result of discrepant interactions between the dorsal and ventral systems in dyslexic adults (van der Mark et al., 2011; Finn et al., 2014; Schurz et al., 2015).

## Conclusion

Reading development is a highly dynamic and individually variable process illustrating an impressive capacity of the brain to accommodate the requirements of a culturally acquired skill. These changes are shaped around the formation of solid associations between dorsal spoken language representations and ventral visual representations that become tuned to written language. Individual differences in the brain’s capacity to accommodate these changes, together with compensatory strengths, such as positive self-beliefs, strong verbal reasoning, vocabulary and attention skills, presumably result in the observed high variability in children’s reading outcome. The observed interactions between the dorsal and ventral reading networks may be best understood from a skill learning perspective involving non-linear developmental changes triggered by the initial acquisition of basic reading skills and their subsequent consolidation with reading practice. Within this framework, variability across typical and dyslexic readers can be characterized by individual learning trajectories with some children facing difficulties especially while learning basic reading skills, including letter-speech sound mappings, and others struggling to make the switch toward consolidating, fine-tuning or generalizing the learned skills and mappings.

One key challenge for the scientific study of reading is understanding which of the observed neuro-behavioral differences between (groups of) readers reflect vulnerabilities or risk factors for developing reading problems versus strengths or compensatory factors, or, especially in older children and adults, consequences of a history of reading problems. Promising experimental paradigms to disentangle these different explanatory possibilities are learning paradigms, such as artificial script learning and text-based recalibration that permit to trace children’s actual learning trajectories. Ideally, these types of paradigms should be combined with a longitudinal multi-level approach incorporating developmental dynamics at various levels including genetic, social environmental, cortical and subcortical brain changes as well as cognitive and behavioral factors (van Atteveldt et al., 2021). This approach ideally integrates developmental changes across both domain-specific and domain-general functional networks, for example, reading, math, and executive function, thereby acknowledging the multi-deficit spectral view of specific learning disorders including dyslexia (Pennington, 2006; van Bergen et al., 2014; Peters and Ansari, 2019). A detailed understanding of children’s learning trajectories across multiple levels and functions starting from the early stages of reading (precursor) skills, will help improve early prediction and, ultimately, prevent the accumulation of reading problems via individualized tailoring of reading support and intervention.

## Author Contributions

All authors listed have made a substantial, direct and intellectual contribution to the work, and approved it for publication.

## Funding

This review is part of a Ph.D. research project funded by the Netherlands Organization for Scientific Research (Vidi-Grant 452-16-004 to MB).

## Conflict of Interest

The authors declare that the research was conducted in the absence of any commercial or financial relationships that could be construed as a potential conflict of interest.

## Publisher’s Note

All claims expressed in this article are solely those of the authors and do not necessarily represent those of their affiliated organizations, or those of the publisher, the editors and the reviewers. Any product that may be evaluated in this article, or claim that may be made by its manufacturer, is not guaranteed or endorsed by the publisher.
